# Characterizing the malaria rural-to-urban transmission interface: The importance of reactive case detection

**DOI:** 10.1371/journal.pntd.0005780

**Published:** 2017-07-17

**Authors:** Karen Molina Gómez, M. Alejandra Caicedo, Alexandra Gaitán, Manuela Herrera-Varela, María Isabel Arce, Andrés F. Vallejo, Julio Padilla, Pablo Chaparro, M. Andreína Pacheco, Ananias A. Escalante, Myriam Arevalo-Herrera, Sócrates Herrera

**Affiliations:** 1 Malaria Vaccine and Drug Development Center, Cali, Colombia; 2 Ministerio de Salud y Protección Social de Colombia, Bogotá, Colombia; 3 Instituto Nacional de Salud de Colombia, Bogotá, Colombia; 4 Department of Biology/Institute for Genomics and Evolutionary Medicine (iGEM), Temple University, Philadelphia- Pennsylvania, United States of America; 5 Caucaseco Scientific Research Center, Cali, Colombia; 6 School of bacteriology and clinical laboratory, Faculty of Health, Universidad del Valle, Cali, Colombia; Common Heritage Foundation, NIGERIA

## Abstract

**Background:**

Reported urban malaria cases are increasing in Latin America, however, evidence of such trend remains insufficient. Here, we propose an integrated approach that allows characterizing malaria transmission at the rural-to-urban interface by combining epidemiological, entomological, and parasite genotyping methods.

**Methods/Principal findings:**

A descriptive study that combines active (ACD), passive (PCD), and reactive (RCD) case detection was performed in urban and peri-urban neighborhoods of Quibdó, Colombia. Heads of households were interviewed and epidemiological surveys were conducted to assess malaria prevalence and identify potential risk factors. Sixteen primary cases, eight by ACD and eight by PCD were recruited for RCD. Using the RCD strategy, prevalence of 1% by microscopy (6/604) and 9% by quantitative polymerase chain reaction (qPCR) (52/604) were found. A total of 73 houses and 289 volunteers were screened leading to 41 secondary cases, all of them in peri-urban settings (14% prevalence). Most secondary cases were genetically distinct from primary cases indicating that there were independent occurrences. *Plasmodium vivax* was the predominant species (76.3%, 71/93), most of them being asymptomatic (46/71). Urban and peri-urban neighborhoods had significant sociodemographic differences. Twenty-four potential breeding sites were identified, all in peri-urban areas. The predominant vectors for 1,305 adults were *Anopheles nuneztovari* (56,2%) and *An*. *Darlingi* (42,5%). One *An*. *nuneztovari* specimen was confirmed naturally infected with *P*. *falciparum* by ELISA.

**Conclusions:**

This study found no evidence supporting the existence of urban malaria transmission in Quibdó. RCD strategy was more efficient for identifying malaria cases than ACD alone in areas where malaria transmission is variable and unstable. Incorporating parasite genotyping allows discovering hidden patterns of malaria transmission that cannot be detected otherwise. We propose to use the term “focal case” for those primary cases that lead to discovery of secondary but genetically unrelated malaria cases indicating undetected malaria transmission.

## Author summary

Malaria is a disease of rural areas in developing countries. Although a rise in urban malaria cases has been noted during the last decade, this trend could be an artifact due to lack of solid data. In order to better understand “urban” and “peri-urban” malaria, we developed a rigorous and systematic methodology that allows characterizing malaria risk in such settings. Our approach is based on cross-sectional studies using active and reactive case detection strategies, genotyping of parasite isolates in order to better understand transmission patterns, and the local assessment of the entomological factors that allow active transmission in urban and peri-urban neighborhoods. This approach was tested in Quibdó, Colombia. No evidence of malaria transmission in urban areas was found. However, we found solid evidence indicating transmission in peri-urban areas due to *Plasmodium vivax* (86%). This was supported by the identification of *Anopheles* mosquitoes and their breeding places. Our results show that reactive case detection is not only an effective strategy to identify cases in areas where transmission is variable and unstable, but also allows the detection of hidden transmission when combined with genotyping methods. Such patterns are undetected by traditional surveillance methods.

## Introduction

Malaria remains a major public health problem that affects 106 countries worldwide mostly in tropical and subtropical regions where ~3.4 billion people are at risk of infection and death [1]. Although malaria is mainly transmitted in rural areas where there are suitable environments for *Anopheles* mosquitoes breeding sites, malaria transmission in urban areas of endemic countries has been increasingly reported over the last three decades [2,3]. Unfortunately, the factors driving urban and peri-urban malaria transmission remain poorly characterized. As urban malaria cases are likely to be found at a broader range of primary care/diagnostic facilities, including hospitals and private laboratories failing to report them to the central surveillance system [4–7], urban malaria control by National Malaria Control Programs (NMCP) requires important administrative changes. Furthermore, there are no clear definitions of “urban”, “peri-urban”, and “rural” settings that properly describe the socioeconomic and ecological contexts where malaria transmission occurs. Thus, there is a need for a rigorous and systematic approach to characterize malaria transmission in the rural-to-urban interface that could provide solid information to assess disease risk in such contexts.

Like other epidemiological settings, urban malaria transmission is influenced by population movements from rural to urban and peri-urban areas. This rural population influx into urban and peri-urban areas facilitates the introduction of malaria from places where the disease is of high prevalence such as those where illegal mining and logging are common[8]. Furthermore, these underserved populations practice subsistence farming and inhabit poor housing with limited access to health services; such social dynamics favor mosquito breeding in areas considered administratively urban [9]. Here, we propose an integrated approach that aims to characterize epidemiologic and entomologic drivers of “urban” and “peri-urban” malarias in settings that are commonly found in Latin America. Our approach was tested in an endemic area of Colombia.

Despite the fact that malaria prevalence is decreasing in Colombia with a 75% reduction in the number of cases since 2000 [1], the National Surveillance System (SIVIGILA) reported an accelerated increase in urban malaria cases from 5.9% in 2011 [9] to 30% in 2015 [10]. Although this increase may be explained by population displacement due to political unrest and illegal crops and mining, there is still the possibility of autochthonous urban transmission. Because only a few studies have focused on urban malaria transmission in Colombia, the growing number of reports on urban malaria cases generates concerns and demands to unequivocally confirm the extent of urban and peri-urban transmission and to establish the corresponding control strategies. Thus, our integrated approach was used to study patterns of malaria transmission in five neighborhoods of Quibdó, the capital of the department of Choco (Colombia)[9,11]. These areas report the greatest number of so called “urban” cases providing an ideal setting for this investigation.

## Methods

### Ethics statement

The study protocol was reviewed and approved by the institutional review board of Caucaseco Scientific Research Center (CECIV, Cali-Colombia) before initiation. Written informed consent (IC) was obtained from each volunteer at enrolment. Parents or legal guardians were asked to consent for children (<18-year-old) to participate in the study, and children older than seven years were asked to sign an informed assent if they wanted to participate. Information obtained from the participants was managed on principles of confidentiality. Immediately after blood sample processing, malaria-positive volunteers were informed and assessed during administration of appropriate anti-malarial treatment at the corresponding point of care. Asymptomatic volunteers did not receive treatment in concordance with the Colombian Ministry of Health (MOH) malaria treatment guidelines.

### Study sites

This study was conducted in Quibdó, which is currently the municipality of Colombia with the highest reported number of malaria cases [10,12]. It is in the Department of Chocó, in the northern area of the Pacific coast in the border with Panama. It has an area of 3,337 km^2^ between the jungle of Darien and the Atrato and San Juan river basins[11]. It consists mostly of a dense tropical rain forest with warm weather (average temperature of 28°C), relative humidity of 90% and an annual rainfall of 8,000–6,000 mm. It has an estimated total population of ~500,000 habitants (2015), with a geographical dispersion 5.4 times higher than the rest of the country [13].

Five sentinel sites (SS) were selected based on location, urbanization and history of malaria cases: La Yesquita, Silencio and Roma which are neighborhoods with urban characteristics located in the center of the city have paved streets, public services and no vegetation close to the houses. On the other hand, Casa Blanca and Cabí are classified as peri-urban, located in the North and South ends of the municipality, respectively, with unpaved streets, variable housing infrastructure, lack of a sewage system and abundant vegetation.

We have considered an urban area as some groups of buildings and contiguous structures grouped in blocks, which are delimited by streets or avenues, with a number of essential services such as aqueducts, sewage, electrical energy, and hospitals and schools. Capital cities and the remaining municipal administrative headings are urban areas. In contrast, a rural area is characterized by the dispersed disposition of houses and agricultural holdings, and a lack of road structure and public services. A peri-urban area is one that combines characteristics both urban and rural, usually located in areas outside the city (DANE (2005) [14]

### Samples

A total of 1mL of blood was collected by venipuncture from every subject, of which ~50 μL were used for thick blood smear (TBS) and the remainder was stored in tubes containing EDTA, refrigerated at 4°C and transported by airplane to the laboratory in Cali for later qPCR analysis and microsatellite (STRs) genotyping. Samples were handled as potential biohazards and all laboratory staff strictly followed bio-safety standardized procedures.

### Study design

We characterized the urban-to-rural malaria interface by integrating epidemiological and entomological approaches. To determine the prevalence of malaria, we first identified eight malaria positive volunteers among individuals seeking diagnosis at the Ismael Roldán hospital in Quibdó. These volunteers reported to live in urban or peri-urban sites of Quibdó and were further selected as sentinel sites (SS) for active case detection (ACD) and reactive case detection (RCD). Then, a cross-sectional survey was performed in five SS from Quibdó, three of them considered urban neighborhoods and two peri-urban. A visit to their neighbourhoods of origin was performed to classify them as urban (2/8) or peri-urban (6/8). For RCD, four houses, closest to the primary case were selected in each neighborhood, using the Vector Born Diseases Program (ETV) census. Eight primary cases identified by ACD were studied with the RCD strategy as well. Men, women and children above one year of age who lived in the household and were present at the moment of the visit were enrolled. A Household was defined as the place where people enrolled in the study lived, including family members, servants, tenants, and others.

Those who agreed to participate answered a symptoms survey and donated a blood sample. Epidemiological questionnaires were answered by the head of the household. A malarial household was defined as one with at least one infected person.

### Sample size

The sample size (n) was calculated for each SS using an estimated prevalence (P) of 2.6% with a confidence level of 95%, 2.5% error (d) according to the following equation n_0_ = (z^α2^), where α = 1.96; P (1—P)/d^2^. Then, it was adjusted according to the population of each neighborhood (N) considering the equation n = n_0_/(1+(n_0_-1)/N)[15].

### Parasite detection

#### Microscopy

Thick blood smears were stained by Giemsa method [16] and examined separately by two experienced microscopists who recorded species and parasite densities, (parasites/200 leukocytes) using 1,000X magnification. A total of 200 microscopic fields were read before recording a negative result.

**Real time quantitative PCR (**qPCR) method based on *Plasmodium* spp. 18S previously described [17], was used for confirmation of parasitemia and parasite species. *P*. *falciparum* and *P*. *vivax* DNA were included as positive control in each qPCR run as well as a DNA extraction negative control. Samples were considered negatives if the threshold cycle was higher than 40. Positive samples were confirmed by an independent DNA extraction and a new round of qPCR by triplicates. Samples were considered positive with at least two positive wells in the confirmation reaction.

**Microsatellite (STRs) genotyping** of genomic DNAs from positive *P*. *vivax* and *P*. *falciparum* samples (n = 29) was performed. Three *P*. *vivax* and two *P*. *falciparum* samples were considered primary cases and 27 secondary cases. Genotyping was performed using fluorescently labeled PCR primers for a set of six selected standardized microsatellite loci for *P*. *vivax* [18]. The following loci were amplified: MS5, MS6, MS15 [19], 8.332, [20], and CLAIN5 and CLAIM10 [21]. Fluorescently labeled PCR products were separated on an Applied Biosystems 3730 capillary sequencer and scored using GeneMarker v2.6.7 (SoftGenetics LLC). After the microsatellite pattern was identified across samples, we scored all the alleles at a given locus if minor peaks were more than one-third the height of the predominant peak. Missing data (no amplifications) were also reported by locus. Single infections were those with only one allele per locus at all the genotyped loci and [20]. Nevertheless, one or more additional alleles at any locus was interpreted as a multiple infection with two or more genetically distinct clones in the same isolate (transmitted by one or several mosquitoes).

### Entomological studies

Collection of adult specimens: Mosquitoes collection was performed using Human Landing Catches (HLC) [22] from 18:00 hours to 6:00 hours in the households of infected volunteers diagnosed by RCD. Collections were carried out simultaneously indoors and outdoors for each house for two consecutive nights. Data on relative humidity and temperature were recorded. Mosquitoes were kept in cups labeled with the date, neighborhood, house code, capture time, mosquitoes quantity and collector's name. Specimens were sacrificed with tri-ethylamine and subsequently individually packaged in 1,5mL vials with a perforated lid, and conserved in airtight bags with silica gel. Technicians in charge of mosquito catches signed an informed consent prior HLC. Adult and immature mosquito specimens were determined using dichotonous keys for *Anopheles* of Colombia [23].

Detection of natural infection with *Plasmodium spp*: After taxonomic identification, mosquitoes’ head and thorax belonging to the same species and capture hour were pooled. Samples were macerated following the MR4 protocol and insert specifications. Circumsporozoite protein (CS) from *P*. *falciparum*, *P*. *vivax* VK-210 and VK-247 variants were detected by Enzyme-Linked ImmunoSorbent Assay (ELISA) using the kit distributed by the Center for Disease Control and Prevention (CDC, Atlanta, USA) [24,25]

Immature collection: We searched SS for open water bodies. The larval habitats search was performed around the households, within a 500-meters radius. Each potential breeding site was georeferenced and characteristics such as size, vegetation type, water type, water body type and water use were recorded. Sampling was carried out using a standard dipper (350 mL) taking ten dips per square meter. Larvae were stored in vials with ethanol for preservation. Each larval container was labeled with date, code, larval number, neighborhood and collector's name.

### Data collection and analysis

Data were recorded in REDCap (v.6.9.4) web application and analyzed using the software R for statistical analysis (v3.3.0) for variables like age, gender, sociodemographic features, living conditions and malaria positive cases, by site and as a total. Non-parametric tests and Chi-square and Fisher’s exact test were performed to check for differences between categories and calculate associations. A level of statistical significance of 5% was used and 95% confidence intervals were calculated for proportions.

The limitations of the study were: A memory bias occurred while conducting the surveys, as for some volunteers it was very difficult to remember exactly the activities carried out weeks before the survey. Volunteers’ displacement was questioned only for the last month. Follow up of some infected cases after diagnosis was difficult because some of them had to leave the city due to their informal jobs”.

## Results

### Demographic features

A total of 717 volunteers (60.0% female) were surveyed using ACD detection in 135 households visited, 58.5% of them located in the peri-urban areas and the rest of them in urban areas. Significant differences were found in sociodemographic variables between urban and peri-urban neighborhoods (Table 1).

**Table 1:** Sociodemographic characteristics by location.

	Subjects	Total (n = 717)	Urban (n = 357)	Peri-urban (n = 360)	p value
		n (%)	n (%)	n (%)	
Sex	Male	287(40.0)	150(42.0)	137(38.1)	
	Female	430(60.0)	207(58.0)	223(61.9)	
Median age (IQR)		25(12–44)	29(18–46)	21(8–38)	<0.0001
Ethnicity	Afro-Americans	557(77.7)	303(84.9)	254(70.5)	<0.0001
	Indigenous	84(11.7)	7(2.0)	77(21.4)	<0.0001
	Mestizo	76(10.6)	47(13.2)	29(8.1)	0.1069
Education level*	Illiterate	110(15.4)	20(5.6)	90(25.1)	<0.0001
	Elementary	215(30.1)	68(19.1)	147(40.9)	<0.0001
	Secondary	244(34.1)	145(40.7)	99(27.6)	0.001
	Technician/University	146(20.4)	123(34.6)	23(6.4)	<0.0001
Occupation (n = 463 Adults)	Housewife	157(33.9)	59(21.5)	98(51.8)	<0.0001
	Merchant	136(29.4)	99(36.1)	37(19.6)	0.0014
	Student	48(10.4)	40(14.6)	8(4.2)	0.0042
	Construction	24(5.2)	15(5.5)	9(4.8)	1.00
	Laborer	19(4.1)	3(1.1)	16(8.5)	0.0015
	Teacher	16(3.5)	14(5.1)	2(1.0)	0.2625
	Unemployed	23(4.9)	14(5.1)	9(4.8)	1.00
	Other	40(8.6)	30(11)	10(5.3)	
		**Houses (n = 135)**	**n = 56**	**n = 79**	**p value**
Housing material**	Brick	66(48.9)	52(92.9)	14(18.2)	<0.0001
	Wood	50(37.0)	4(7.1)	46(59.7)	<0.0001
	Shack	17(12.6)	0(0)	17(22.1)	0.0017
Water source**	Rain water	83(61.5)	9(16.1)	74(96.1)	<0.0001
	Aqueduct	37(27.4)	37(66.1)	0(0)	<0.0001
	River or well	13(9.6)	8(14.3)	5(6.5)	0.6362
Public services	Electricity	104(77.0)	56(100)	48(60.8)	0.1226
	Sewage system	38(28.1)	38(67.9)	0(0)	<0.0001
	Garbage collection	54(40)	50(89.3)	4(5.1)	<0.0001

*Two people did not indicate education level

**Two people answered other type of housing material or water source respectively

Median ages were significantly higher in urban neighborhoods. Overall the Afro-American ethnic group was predominant (77.7%), indigenous population was significantly higher in both areas peri-urban areas (21.4%); especially in Cabí, where 91.6% of the population share this ethnic origin. Education level was significantly lower in peri-urban areas, with 25.1% of the population being illiterate. Most frequent occupations were merchant in urban areas and housewife in peri-urban areas.

Housing conditions were also significantly different (Table 1). In the urban neighborhoods, most houses were made of brick, 66.1% had aqueducts and the majority of them had access to electricity (100%), garbage collection system (89.3%) and sewage system (67.9%). In the peri-urban neighborhoods, the predominant housing material was wood (59.7%), and none of the houses had an aqueduct service or sewage system. Water supply was obtained from rain in 74 of 79 houses while the remaining obtained water directly from the river or had a well. Only three houses in Casa Blanca and one in Cabí had a garbage collection system.

### Malaria cases by passive case detection

Eight primary cases were recruited in a hospital in Quibdó where they attended to seek diagnosis. Four of them were caused by *P*. *vivax*, three by *P*. *falciparum* and one was a mixed infection. Two cases came from urban sites and the other six from peri-urban neighborhoods. Most of the cases were in Afro-descendants, with complete to incomplete secondary education and with informal jobs. Five of the eight cases were men.

### Malaria prevalence by active case detection

**Fig 1:**
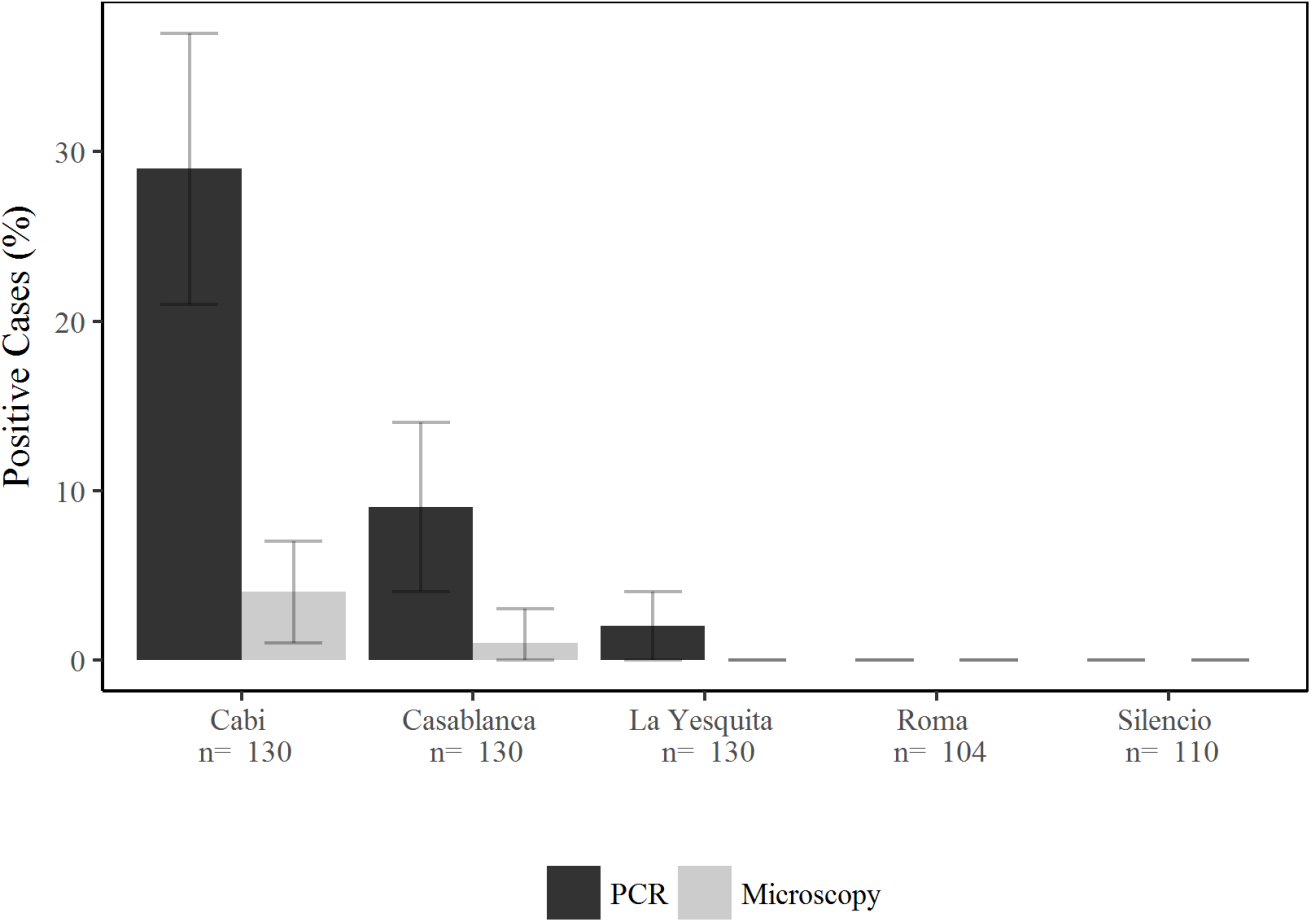
Malaria prevalence by PCR and Microscopy found by active case detection.

The overall prevalence of malaria by ACD was 1% (6/604) using microscopy and 9% (52/604) by qPCR. A total of 44 cases (85%) of the 52 detected by qPCR were due to *P*. *vivax* being the predominant species. Ninety-six percent of the detected infections were in peri-urban areas, presenting a significant difference with those originated in urban neighborhoods (p<0.0001). Cabí was the neighborhood with the highest prevalence (29% ±8 SE), followed by Casa Blanca (9% ±5 SE qPCR). In the urban neighborhoods, only two submicroscopic infections were diagnosed (Fig 1).

### Reactive case detection

Eight primary asymptomatic infections from ACD were selected for RCD, six for *P*. *vivax* and two for *P*. *falciparum*. During the RCD, 33 houses were studied, 18 in Cabí, 10 in Casa Blanca and five in La Yesquita, and a total of 113 volunteers were surveyed. Twenty-seven secondary cases (three by TBS and 27 by qPCR) were identified, all of them from peri-urban areas. The overall percentage of positives among the screened people was 2.7% by microscopy and 24% by qPCR. A total of 13 malarial houses were found.

In addition, a second round of RCD was performed around the eight symptomatic cases recruited by PCD. A total of 175 volunteers among family members and neighbors were included. Fourteen secondary infections were detected (eight by TBS and 14 by qPCR), representing an infection rate of 4.6% by microscopy and 8% by PCR. Twelve cases were caused by *P*. *falciparum* and the other two by *P*. *vivax*.

Twelve of the 14 secondary cases were female, most of them Afro-descendant, four housewives, three students and one with another type of job. Seven were children under eleven years, 7/14 were older than 17 years, one of them 47 years old. One 10-year-old child was an asymptomatic positive case by PCR from an urban site, he did not have recent history of recent displacement outside Quibdó but had moved to other peri-urban neighborhood close to his home. A total of 10 malarial houses were detected in this survey.

In total, using the RCD strategy we found 41 malaria cases, 27 by *P*. *vivax* (66%) and 14 *P*. *falciparum* (34%) and 22 malarial houses. No mixed infections were detected.

### Malaria clinical manifestations

A high number of asymptomatic volunteers were identified, i.e. 58 of the 93 cases diagnosed by qPCR did not report any symptoms at the time of blood examination and none reported to have had malaria symptoms for the last 15 days. Asymptomatic infection was more frequent with *P*. *vivax* (46/71). Of the nine volunteers with infection diagnosed by microscopy, seven presented with symptoms. Thirty-five cases showed symptoms; the most common symptoms were fever (25/35), headache (25/35), chills (11/35), muscles pain (9/35), malaise (6/35), and profuse sweating (3/35).

### Microsatellite (STRs) genotyping

**Fig 2:**
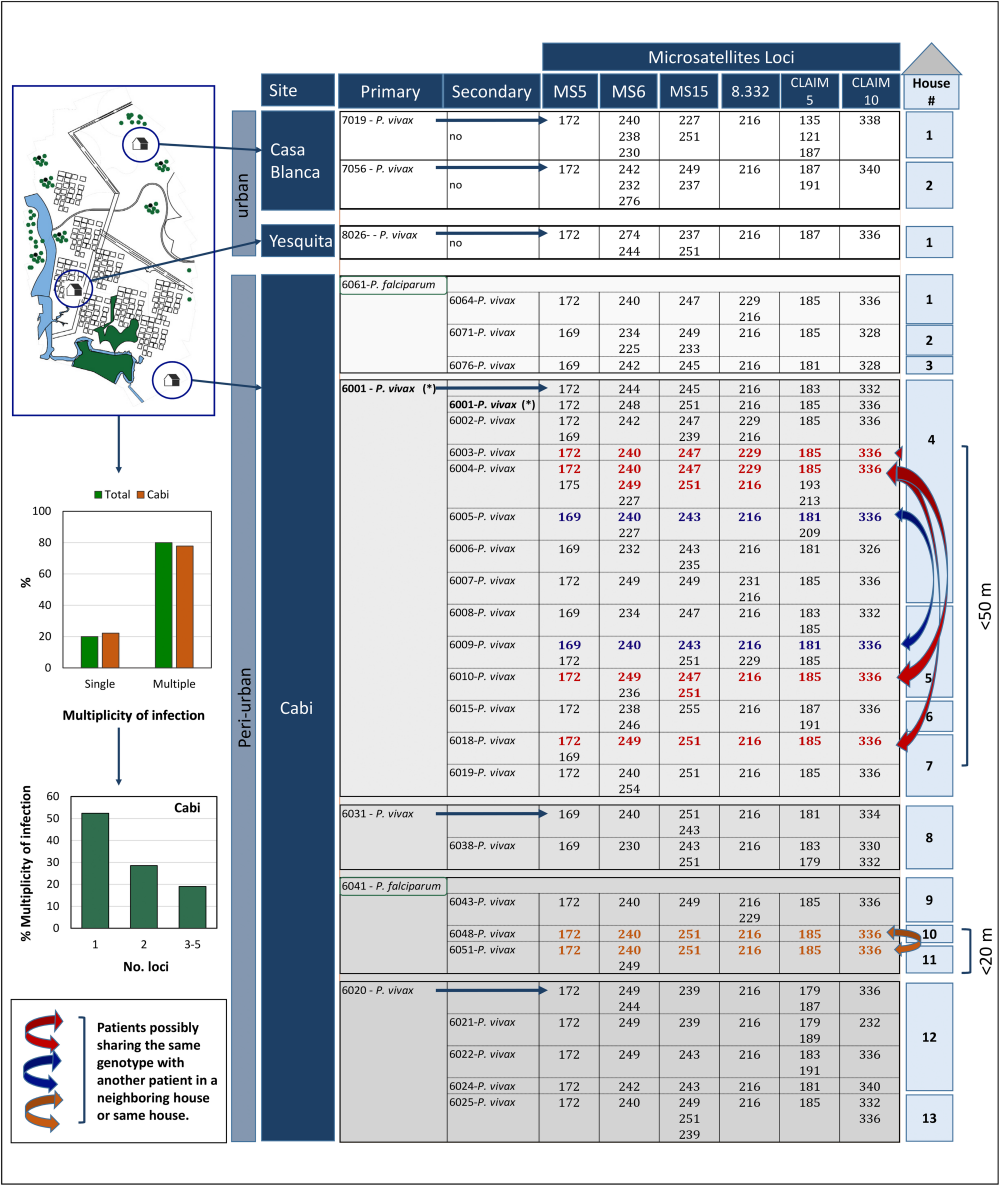
Parasite genotyping. The map that appears in this figure was made by a graphic designer of our Caucaseco Scientific Research Center Cali, Colombia, using the program AutoCAD.

Out of 8 primary cases (6 *P*. *vivax* and 2 *P*. *falciparum*), 27 secondary cases were detected in Cabí: 21 multiple infections (>1 allele in at least 1 microsatellite locus) and only 6 single infections for the set of microsatellite loci used. These multiple infections included 11 (52.4%) with 2 alleles in at least 1 locus, 6 (28.6%) with at least 2 alleles in 2 loci, and 4 (19%) with > 2 alleles in 3 or more loci (Fig 2)[26].

None of the genotypes found in the primary cases matched those found in the secondary cases. Furthermore, two primary cases were *P*. *falciparum* but all the detected secondary cases were *P*. *vivax*. This pattern indicates that the secondary cases were not related to the primary cases that allowed their detection. Nevertheless, some genotypes were shared among the secondary cases. Specifically, related genotypes were found in two patients that inhabited the same house (4), as well as patients that inhabited houses that were near (4 and 7 separated by ~43 mts, 4 and 5 separated by ~13 mts, and 204 and 207 separated by ~18 mts) (Fig 2).

### Entomology studies

Entomological studies were performed in 15 houses where at least one malaria case was detected. These houses were distributed in three SS, one urban (La Yesquita) and two peri-urban (Casa Blanca and Cabí). Neither mosquitoes nor breeding sites were found in the urban setting. On the contrary, both peri-urban areas registered the presence of immature and adult *Anopheles* mosquitoes.

#### Adult mosquito collections

A total of 1,305 *Anopheles* mosquitoes belonging to four species were collected. In Casablanca, *An*. *darlingi* (n = 365) was the most abundant species (69.5%) followed by *An*. *nuneztovari* (27.4%), *An triannulatus* (2.9%) and *An*. *apicimacula* (0.2%). In contrast, only two species were registered in Cabí (n = 780): *An*. *nuneztovari* (75.6%) and *An*. *darlingi* (24.4%) (Table 2).

**Table 2:** Number of adults and larvae mosquitoes collected for each neighborhood.

	Cabi			Casablanca			La Yesquita			Total	
	Indoors	Outdoors	Larvae	Indoors	Outdoors	Larvae	Indoors	Outdoors	Larvae	Adults	Larvae
***An*. *apicimacula***	0	0	0	0	1	0	0	0	0	1	0
***An*. *darlingi***	62	128	2	148	217	5	0	0	0	555	7
***An*. *nuneztovari***	201	389	26	63	81	6	0	0	0	734	32
***An*. *triannulatus***	0	0	0	6	9	0	0	0	0	15	0
**Total**	263	517	-	217	308	-	0	0	-	1305	39
**Total by Neighborhood** (percentage collected)	780 (59.7%)		28 (71.8%)	525 (40.3%)		11(28.2%)	0		0		
**No Houses/Breeding Sites**	11		13	3		11	1		0	15	24

**Fig 3:**
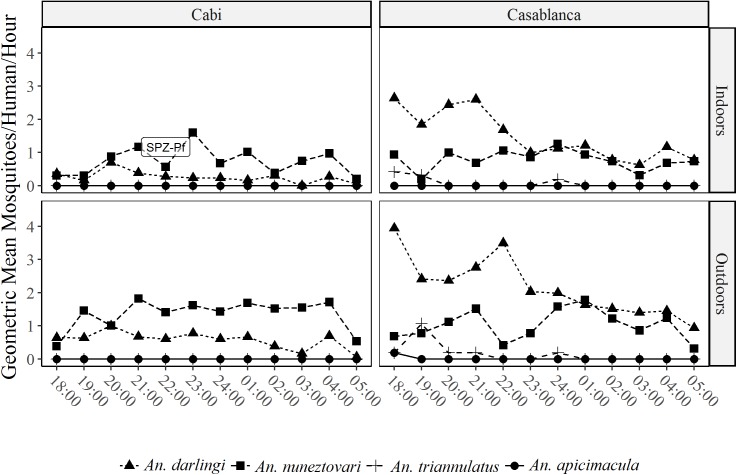
Biting behavior of adult mosquitoes.

Biting behavior of the most abundant species differed between species and areas. In Casa Blanca, *An*. *nuneztovari* is active during the whole night without distinct activity peaks, being equally active indoors and outdoors (Fig 3).

The average rate for biting activity in this locality was 10.94 bites per person per night. The biting activity rate of *An*. *nuneztovari* and *An*. *darlingi* was 3.00 and 7.60 bites per person per night, respectively. On the other hand, in Cabí the biting activity of *An*. *darlingi* was more intense in the first hours of the night (18:00 to 23:00 hours) and no differences in density values were found in indoors and outdoors captures. Average rate for biting activity in Cabí was 1.48 bites per person per night being the biting activity rate for *An*. *nuneztovari* 1.12 and for *An*. *darlingi* 0.36 bites per person per night.

### Natural infection of mosquitos with *Plasmodium* spp.

Natural infection by *P*. *falciparum* and *P*. *vivax* (VK-210 and VK-247 variants) was analyzed in 1,305 adult *Anopheles* mosquitoes; *An*. *nuneztovari* (n = 734), *An*. *darlingi* (n = 555), *An*. *triannulatus* (n = 15) and *An*. *apicimacula* (n = 1). One *An*. *nuneztovari* obtained in Cabí was found infected with *P*. *falciparum* (1/590), corresponding to an infection rate of 0.17% in this locality. This infected mosquito was captured biting indoors between 22:00h and 23:00h in the Urada indigenous community.

### *Anopheles* immature collections

A total of 24 open water bodies were examined and georeferenced. From these, four were positive for *Anopheles* mosquito larvae in Casa Blanca and six in Cabí. Of the ten positive larval habitats, seven were excavation site type, two were stream and one was a puddle. A total of 100 larvae were collected in the breeding sites from these 40 late (3rd and 4th) instar larvae were identified belonging to the two most abundant species. *An*. *nuneztovari* was found in excavation sites and *An*. *darlingi* in streams and puddles (Table 2).

## Discussion

Our integrated approach shows a complex pattern of malaria transmission in the rural-to-urban interface in this Colombian community. Although all the selected SS were administratively classified as urban neighborhoods, they showed significant differences in terms of sociodemographic characteristics, prevalence of *Plasmodium* species, and distribution of the different vector species. Based on the criteria developed here, we can unequivocally classify three SS (La Yesquita, Roma and Silencio) as urban. However, the others (Cabí, Casa Blanca) should be considered peri-urban/rural settings. This highlights the need to reach a consensus for the administrative classification of the territory. Social and ecological characteristics of each studied neighborhood would be most relevant for an accurate classification as well as in terms of designing malaria control programs.

Our results also show that malaria continues to be a disease that mainly affects vulnerable communities. Indeed, 97.5% of the cases were diagnosed in peri-urban areas inhabited by young and less educated people living in precarious conditions. The significant greater prevalence of malaria among children could be partially explained by the age structure of the population [1]. Lower education levels have also been associated with higher malaria risk [27]. In terms of occupations, 78.6% of the adult volunteers were housewives, unemployed population, students and merchants. This finding supports the hypothesis that infection is occurring mostly in homes rather than work places, reducing the possibilities of bias related to occupational risks.

As expected, the number of cases detected by PCR (93 cases) was significantly higher (5.6 times) than that detected by microscopy (16 cases), strengthening the need to re-evaluate the diagnosis methods used in this type of epidemiological settings. The confirmed presence of submicroscopic infections represents an important public health problem, as the unidentified positive cases will not receive treatment, and will keep contributing to transmission maintenance [28]. Therefore, it is of paramount importance to accurately determine parasite prevalence for monitoring malaria interventions.

*P*. *vivax* was the predominant species and most cases were diagnosed in Cabí, where the population was predominantly indigenous. This could be explained by their Duffy positive (Fy+) genotype, which makes this population susceptible to *P*. *vivax* infection [29] as compared to the Afro-descendant population which in this region display a Fy- prevalence of 38.9% [30].

This investigation also shows that RCD combined with parasite genotyping allows a better assessment of the transmission patterns which are far more complex than the ones inferred from epidemiologic data alone [31,32]. RCD usually involves detection of a primary case that is further referred to as “index case”. This term is commonly used in the context of infectious diseases to denote a first detected case that allows the identification of secondary cases that are usually part of the same transmission tree. Loosely used, however, a primary case can simply indicate an environment that can sustain transmission where secondary cases are spatially clustered. Thus, in such context, the epidemiological evidence does not make a distinction between a cluster of cases that is the result of a recent introduction or simply ongoing transmission that has not been detected [32,33]. Considering that RCD is laborious in areas of low transmission [32], the incorporation of parasite genotyping is essential to increase the information yield by RCD. It allows separating between recently introduced cases [34,35] and hidden ongoing transmission that simply remains elusive to the control program. Such distinction is of importance during the elimination phase or wherever control measures should be deployed to control malaria in urban or peri-urban settings. This investigation indicates that, in the context of these study sites, the primary case detected areas with ongoing transmission, but secondary cases were not part of the same transmission tree as expected under a scenario of recent introduction. Indeed, two primary cases were caused by *P*. *falciparum* and all the secondary were *P*. *vivax* (Fig 2). This indicates that the social and environmental conditions in the study areas can sustain asymptomatic-submicroscopic malaria patients that could allow for the re-emergence of malaria whenever control strategies are relaxed or environmental conditions change [36].

Based on the patterns detected in this investigation, we propose to reserve the use of the term index case for those primary cases that allow the detection of secondary cases that are part of the same genetically related cluster indicating a single (or few) reintroductions. Accordingly, we propose using the term ‘focal case’ to denote a primary case that allows for the identification of hidden (ongoing) malaria transmission as evidenced by a cluster of unrelated secondary cases. At least in the context of *P*. *vivax*, we could also use the proportion of multiple infections as a metric for ongoing transmission since those indicate superinfections [37]. In this context, patients with complex genotypes (multiple alleles in >2 loci, see [26]) may indicate two or more infectious bites providing additional evidence of stable undetectable transmission.

Previous studies in Quibdó have shown that there are malaria transmission hot spots, which are located in peri-urban areas, and that neighborhoods such as Cabi in the South and Casa Blanca in the North are areas with a high incidence and prevalence of malaria, as confirmed in the present study. It would be important to follow these transmission hotspots with active surveillance and RCD, including molecular diagnostic methods to properly assess the effect of interventions on malaria transmission. These interventions should be evaluated to confirm its efficacy in terms of reducing morbidity and mortality associated with malaria transmission. The importance of this hidden malaria transmission is evidence by the fact that Chocó significantly contributed to the malaria rebound that occurred in Colombia during 2016, with an expansion of 62% in the number of cases, of those, 16% were reported from Quibdó [10,38].

Finally, a worldwide effort to understand urban malaria has been undertaken by the International Centers of Excellence for Malaria Research-ICEMRs. In a recent publication authored by this group, one of the pillars to this understanding includes the definition of what constitutes “urban” and “peri-urban” malaria. There is no global consensus definition of urban malaria and most countries use an administrative definition [4]. In Colombia, it would be important to redefine the concept of "administrative urban municipality", which includes urban and peri-urban areas in terms of the charts used by the notification system (SIVIGILA), to classify reported cases correctly.

### Conclusions

No evidence for urban malaria transmission was found in Quibdó. The cases found in urban areas were imported from other cities or peri-urban neighborhoods with high prevalence. Thus, malaria transmission is mainly peri-urban, and autochthonous transmission occurs mainly in indigenous communities. The implementation of RCD with molecular diagnostics and genotyping, allow the detection of hidden malaria transmission clusters. This approach is suitable to better understand the efficacy of the malaria control programs interventions.
